# Pre-operative knee extensor and flexor torque after secondary ACL rupture: a comparative retrospective analysis

**DOI:** 10.1186/s13102-022-00531-0

**Published:** 2022-07-19

**Authors:** Marlene Mauch, Ramona Ritzmann, Christophe Lambert, Markus Wenning, Clara Ebner, Leonie Hartl, Albrecht H. Heitner, Jochen Paul, Christoph Centner

**Affiliations:** 1 Rennbahnklinik, Muttenz, Switzerland; 2 Department of Trauma and Orthopaedic Surgery, University of Witten/Herdecke, Cologne Merheim Medical Centre, Cologne, Germany; 3 Department of Orthopedic and Trauma Surgery, Medical Faculty, University Medical Center, University of Freiburg, Freiburg, Germany; 4 Department of Sport and Sport Science, University of Freiburg, Schwarzwaldstraße 175, 79117 Freiburg, Germany

**Keywords:** ACL revision, ACL re-rupture, Strength, Pre-rehabilitation, Isokinetic

## Abstract

**Background:**

Secondary anterior cruciate ligament (ACL) ruptures are a relevant clinical concern after surgical treatment of a primary ACL rupture. However, there is a lack of scientific evidence related to the role of muscle strength prior to revision surgery in a second ACL rupture. The aim of this study was to assess differences in knee extensor and flexor strength in patients *before* primary and secondary ACL reconstruction compared to healthy controls.

**Methods:**

In total, *n* = 69 age, weight and sex matched individuals were included in the study: *n* = 23 patients with isolated primary ACL rupture, *n* = 23 with secondary ACL rupture, and *n* = 23 matched healthy controls. Maximal isokinetic knee extension and flexion torque normalized to body mass was assessed for both legs.

**Results:**

For patients with secondary ACL ruptures, torques were reduced in the non-injured (extension: 1.94 Nm/kg vs. 2.46 Nm/kg, *p* < 0.05, flexion: 1.25 Nm/kg vs. 1.59 Nm/kg, *p* < 0.05) and the injured leg (extension: 1.70 Nm/kg vs. 2.46 Nm/kg, *p* < 0.05, flexion: 1.14 Nm/kg vs. 1.59 Nm/kg, *p* < 0.05) compared to healthy controls. For patients with a primary ACL rupture torques were reduced in the non-injured (extension: 1.92 Nm/kg vs. 2.46 Nm/kg, *p* < 0.05, flexion: 1.24 Nm/kg vs. 1.59 Nm/kg, *p* < 0.05) and the injured leg (extension: 1.38 Nm/kg vs. 2.46 Nm/kg, *p* < 0.05, flexion: 1.01 Nm/kg vs. 1.59 Nm/kg, *p* < 0.05) compared to healthy controls. There were no differences between patients with primary and secondary ruptures, except of the knee extension on the injured leg showing higher values after a secondary ACL rupture (1.38 Nm/kg vs. 1.70 Nm/kg, *p* < 0.05).

**Conclusions:**

The findings indicate that maximal knee torques were significantly reduced in patients with primary and secondary ACL ruptures before surgical reconstruction for the non-injured and injured leg as compared to healthy controls. Further investigations are needed to assess strength abilities before and after a second revision within a prospective design.

## Background

Approximately 200,000 anterior cruciate ligament (ACL) ruptures occur in the United States annually, with an incidence of 36.9–68.6 per 100,000 person-years [1–3]. Pivoting and cutting sports, as well as female sex are well described risk factors of suffering ACL tears [4, 5]. For the treatment of ACL ruptures, both conservative (*e.g.,* physical rehabilitation) and surgical treatments are currently recommended depending on the demands on knee stability, age and patients’ expectation in athletic performance [1–3]. Generally, approximately 90% of patients experiencing an ACL rupture undergo ACL reconstruction using various graft sites [6].

For a safe return to sport (RTS) after ACL reconstruction, current guidelines recommend a criterion-based rehabilitation of 6–9 months with a need of individual adjustments to the patient’s characteristics and functional abilities [7]. However, despite strict rehabilitation protocols a previous study reports re-injury rates being as high as 29.5% within 2 years following RTS [4]. Secondary ACL ruptures therefore remain a relevant problem [8]. Key risk factors for re-injury after ACL reconstructions are younger age [9, 10], higher activity level or an early return to high level sporting activities [4, 10–12].

Both primary as well as secondary ACL ruptures are accompanied by severe neuromuscular and myo-structural consequences resulting in pronounced decrements in proper functioning [13, 14], performance and balance between knee extensor and flexor muscles [15]. De Jong and colleagues, for example, demonstrated that side-to-side quadriceps strength is still ~ 20% compromised even 1-year after ACL reconstruction [16]. Compared to healthy controls, these strength deficits were even higher (~ 25–26%) [17]. Primary cause of muscle weakness following ACL injury include muscle atrophy, arthrogenic muscle inhibition and voluntary activation failure [13–15, 18]. Given that in a healthy and ACL reconstructed knee the ACL serves as the primary restraint to anterior translation of the tibia relative to the femur [19], there is a need to focus on the knee muscles as important stabilizers of the joint [20–22]. It has been shown that the muscle strength of knee extensors and flexors is a multiple of the resistance of the original ACL, autograft or allograft of various sides [23, 24] and therefore is an active prerequisite that serves to protect the knee joint against damage [25].

In addition to long-term consequences of primary and secondary ACL ruptures, recent research interests have shifted towards evaluations of strength factors *prior to* ACL reconstruction [26]. Interestingly, several studies have demonstrated that strength levels measured prior to reconstruction surgery are predictive of long-term outcomes such as knee function [27, 28] or post-operative strength [29] in patients with unilateral primary ACL rupture. Although such deficits have been reported in patients with a primary ACL rupture, evidence regarding pre-operative strength deficits are missing in patients with a secondary ACL rupture.

The aim of this study was to assess differences in knee extensor and flexor strength in patients *before* primary and *before* secondary ACL reconstruction surgery in the injured and non-injured leg as compared to healthy controls. It was hypothesized, that the strength of the non-injured leg of patients with isolated primary or secondary ACL rupture would be comparable to the strength of a matched healthy control group. Further the strength of the injured leg as well as of the non-injured leg after a second injury would be worse than after the primary injury due to a previous ACL reconstruction surgery.

## Methods

### Study design

A retrospective cohort study design was implemented. Therefore, patients with secondary ipsilateral ACL rupture were compared to patients with primary ACL rupture and healthy controls. All study procedures were conducted in accordance with the latest version of the Declaration of Helsinki and the study was approved by the local ethics committee (EKNZ 2021–01,106). The need for an informed consent was waived by the ethics committee (Ethikkommission Nordwest- und Zentralschweiz) due to the retrospective nature of the study design.

### Participants

Patient records of *n* = 120 patients with primary and *n* = 120 patients with secondary ACL rupture between the years of 2015 and 2019 were consecutively recruited in the medical consultations of an orthopedic clinic and screened in the biomechanical laboratory of the clinic. Patients were included in the analysis if they were aged between 15 and 66 years and experienced an isolated ACL injury for both primary and secondary ACL ruptures. Exclusion criteria for both primary and secondary ACL ruptures were: (1) any additional lesion of the meniscus, (2) any additional lesion of knee cartilage structures, (3) any additional lesion of the medial or lateral knee ligaments, (4) any lesion of the bone, (15) any further knee surgeries (such as meniscal suture, axis corrections, cartilage interventions). For patients with a secondary ACL rupture the inclusion criteria were in addition: (1) the time period between the reconstruction surgery for the primary ACL rupture and the secondary ACL rupture was > 1 year, (2) the surgery of the primary ACL rupture was without complications (e.g. infection, healing, nerve damage) (3) the rehabilitation period after the primary ACL surgery was performed according to evidence-based rehabilitation regimens and completed according to standardized return-to-sport criteria [30]. Primary injury mechanisms included landing and cutting maneuvers during sports. All patients from the primary and secondary ACL groups were operated in the same clinic. Healthy individuals without any injuries on the lower extremity were recruited from the surrounding area based on an advertisement. Anthropometric, medical as well as torque data were independently extracted by two researchers, with each patient receiving a unique pseudonymized identification number.

### Torque measurements

Maximal concentric knee extension and flexion torques were measured using an isokinetic dynamometer (Humac®/NormTM, CSMi, Stoughton, Massachusetts, US). Reliability and validity of this dynamometer and procedure are described elsewhere [31]. Participants were seated in a rigid chair and firmly strapped at the thorax, hip and distal thigh [32]. The trunk was leaning at 85° against the back rest. The rotational axis of the dynamometer was aligned to the lateral femoral epicondyle, and the lower leg was attached to the dynamometer lever arm above the medial malleolus, with no fixation of the ankle joint. The measurements were preceded by 10 min of warm-up at a stationary cycle ergometer (50 W) [33], followed by six submaximal familiarisation trials in the isokinetic dynamometer [26].

For data assessment the protocol by Li and colleagues [31] was used with concentric-concentric contractions a 60°/s angular speed, in the full individual range of motion (ROM) due to its high test–retest reliability [31]. Two sets of five repetitions with maximum effort were executed. Both legs were tested separately and each trial was initiated with the non-injured limb. Between sets, patients had at least rest for 1 min [34]. Outcome parameters were: maximal knee extension and flexion torque normalized to body mass for the injured and non-injured leg for patients with an ACL rupture [26]. Normalization was applied to avoid inhomogeneous results and achieved throughout division of the torque by the body mass (Nm/kg) for each individual [35]. For healthy controls, values for the left and right legs were averaged for comparisons with the injured and non-injured legs from the primary and secondary injured participants.

### Statistics

All statistical analyses were completed using R software [36] and figures were created using the ggplot package [37]. After checking for normal distribution (Shapiro–Wilk test) and homogeneity of variances (Levene’s test), a one-way ANOVA was performed. In case of significant findings in the ANOVA, Benjamini–Hochberg corrected post-hoc tests were calculated. All data is presented as mean ± standard deviation, if not indicated otherwise. The alpha level was set to *p* < 0.05 and effect sizes were calculated using partial eta-squared (*η*^2^).

## Results

**Fig. 1:**
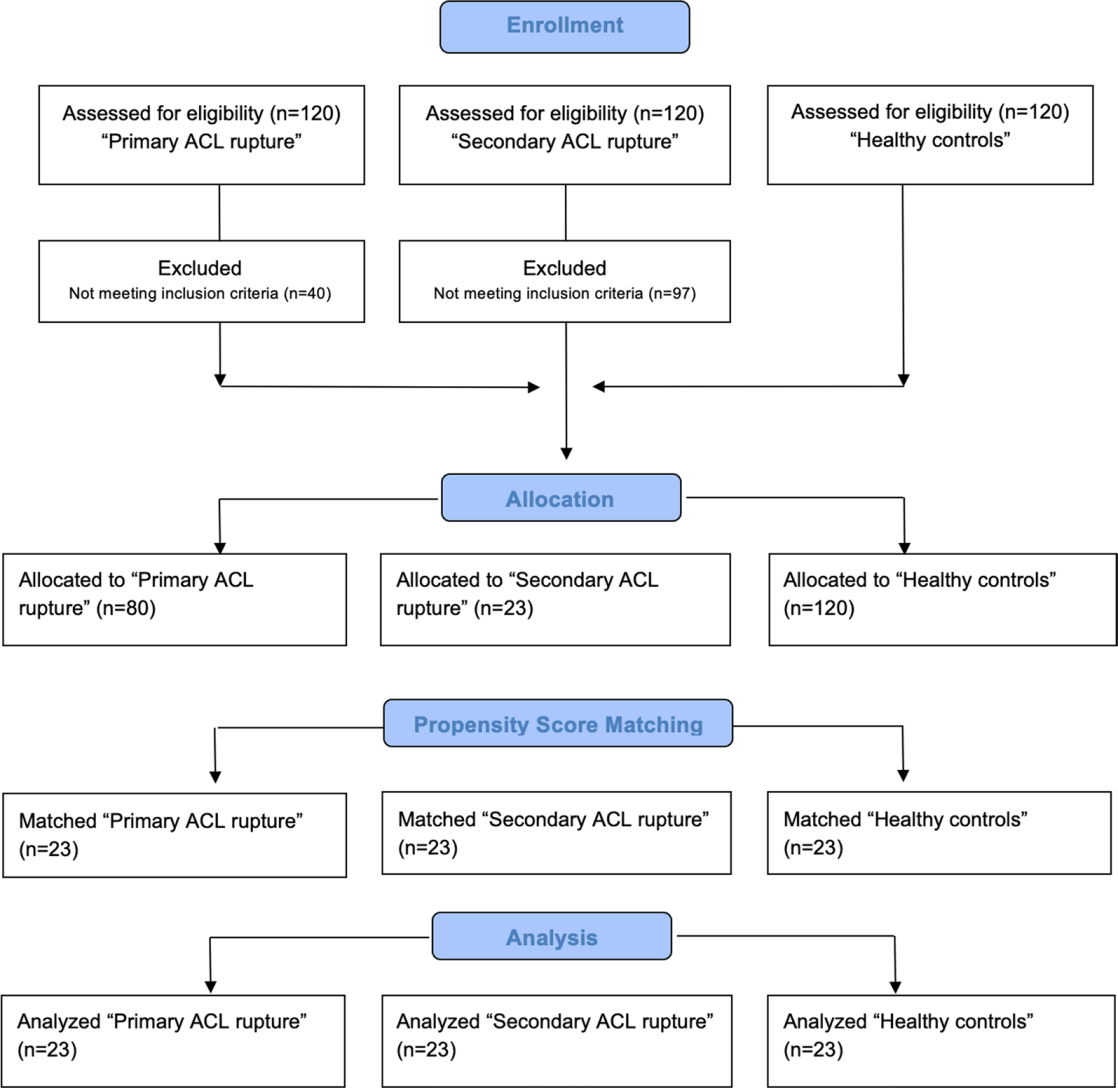
Flow chart diagram—description of the study population

After screening, *n* = 23 patients with isolated secondary ACL rupture remained. These individuals were then matched with an equal number of patients with primary ACL rupture and healthy controls by (1) weight, (2) height, (3) BMI, (4) age and (5) sex, using nearest neighbor propensity score matching [38]. Fitness and activity levels were comparable between groups as all participants underwent identical rehabilitation protocols 9 months after the surgical treatment [26] (Fig. 1). In total, *n* = 69 individuals were included in the final analysis with *n* = 23 individuals in each group (healthy vs. primary ACL rupture vs. secondary ACL rupture). Baseline characteristics are presented in Table 1. No significant differences were observed for any anthropometric variable (*p* > 0.05). For patients with a secondary ACL rupture the time period between the reconstruction surgery of the primary ACL rupture and the secondary ACL rupture laid between1 and 7 years (1010.0 ± 727.3 days).

**Table 1:** Participant characteristics (*n* = 69)

Group	Sex	Height	Weight	BMI	Age	Time interval between injury and data assessment (d)
Healthy	f = 7; m = 16	178.4 ± 8.3	77.7 ± 11.0	24.4 ± 2.3	28.7 ± 8.9	N/A
Primary ACL rupture	f = 7; m = 16	177.3 ± 9.7	76.0 ± 13.1	24.0 ± 2.6	25.3 ± 6.7	18.2 ± 9.0
Secondary ACL rupture	f = 8; m = 15	173.2 ± 10.5	74.2 ± 14.7	24.5 ± 3.2	26.1 ± 7.0	16.8 ± 10.7

### Non-injured leg

#### Knee extension torque

**Fig. 2:**
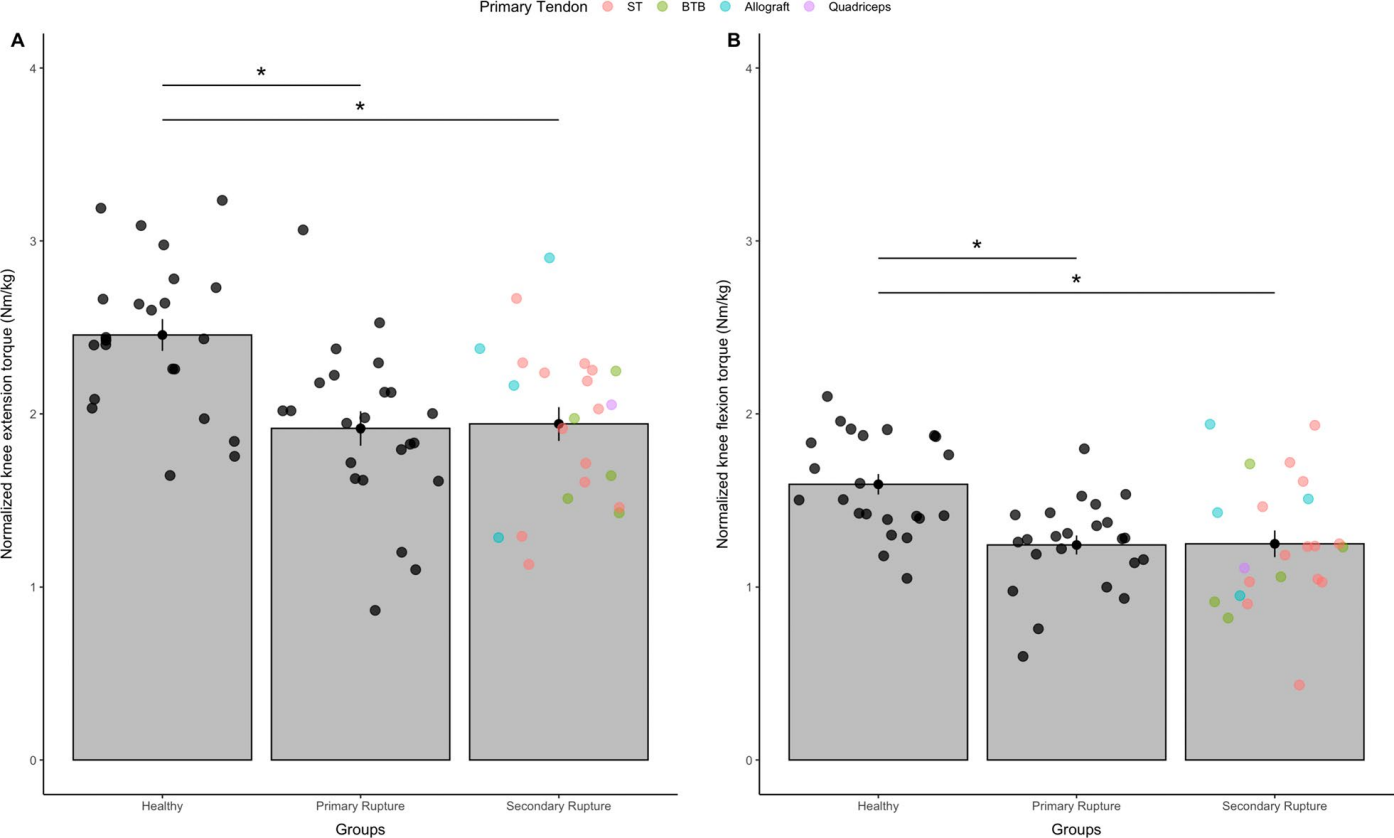
Pre-operative normalized knee extension **A** and flexion **B** torque (Nm/kg body weight) of the ***non-injured*** leg. Bars show the mean and error-bars standard error. Points indicate the tendon type (*n* = 69). Filled and colored dots represent primary tendon grafts; * indicates significant differences following one-way ANOVA (*p* < 0.05); BTB = bone-to-bone, ST = Semitendinosus, BW = body weight

Before surgery, patients suffering from a primary ACL rupture demonstrated normalized knee extension torque levels of 1.92 ± 0.47 Nm/kg (146.5 ± 47.6 Nm) and patients with a secondary ACL rupture torques of 1.94 ± 0.46 Nm/kg (143.4 ± 44.9 Nm) at the non-injured leg, respectively. In contrast, knee extension torques of 2.46 ± 0.44 Nm/kg (192.2 ± 49.7 Nm) were observed in the healthy control group. After calculation of a one-way ANOVA, significant group differences were found (*F*_(2, 66)_ = 10.1, *p* < 0.01, *η*^2^ = 0.234) (Fig. 2A), with lower values in both ACL revision groups compared to the healthy control group (*p* < 0.01). No significant differences were detected between patients with a primary as compared to patients with a secondary ACL rupture (*p* = 0.85).

#### Knee flexion torque

Regarding knee flexion torque, patients with primary ACL ruptures presented normalized torque levels of 1.24 ± 0.26 Nm/kg (94.0 ± 25.2 Nm), whereas patients with secondary ACL ruptures demonstrated torque levels of 1.25 ± 0.37 Nm/kg (92.7 ± 31.3 Nm). In the healthy control group, knee flexion torques of 1.59 ± 0.28 Nm/kg (125.0 ± 32.3 Nm) were found.

After statistical analysis, results from ANOVA revealed significant group differences (*F*_(2, 66)_ = 9.62, *p* < 0.01, *η*^2^ = 0.226) (Fig. 2B) with significantly higher torques in the healthy control group compared to both patients with a primary (*p* < 0.01) and patients with a secondary ACL rupture (*p* < 0.01). Again, no significant differences were found between primary and secondary ACL patients (*p* = 0.942).

### Injured leg

#### Knee extension torque

**Fig. 3:**
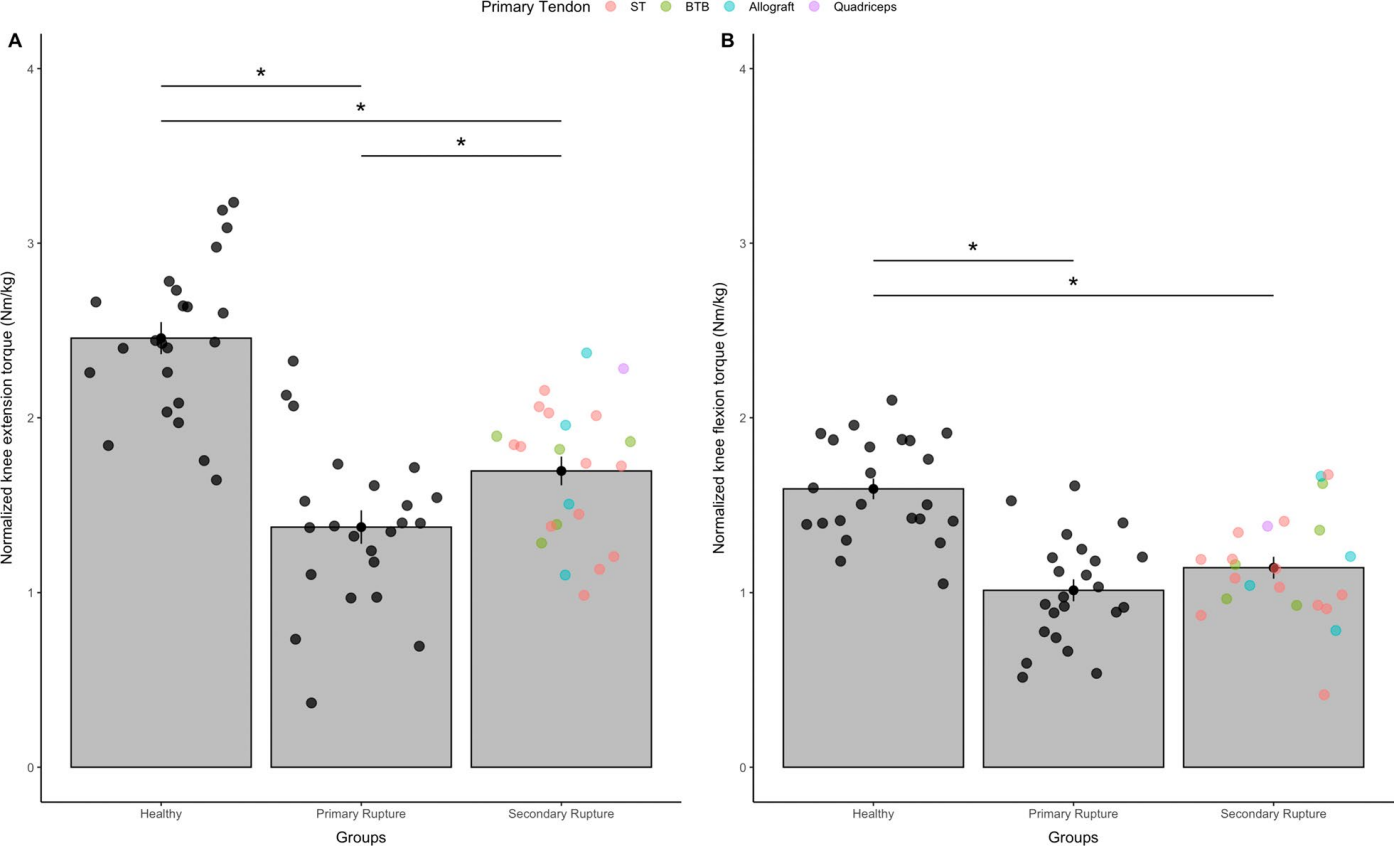
Pre-operative normalized knee extension **A** and flexion **B** torque (Nm/kg body weight) of the ***injured leg***. Bars show the mean and error-bars the standard error. Points indicate individual data points (*n* = 69). Filled and colored dots represent primary tendon grafts; * indicates significant differences following one-way ANOVA (*p* < 0.05); BTB = bone-to-bone, ST = Semitendinosus, BW = body weight

In the injured leg, patients with a primary ACL rupture demonstrated pre-operative normalized knee extension torques of 1.38 ± 0.46 Nm/kg (104.0 ± 41.0 Nm) and patients with a secondary ACL rupture torques of 1.7 ± 0.39 Nm/kg (127.5 ± 44.0 Nm), respectively. In healthy controls, knee extension torque levels of 2.46 ± 0.44 Nm/kg (192.2 ± 49.7 Nm) were observed. Following statistical analysis, significant group differences (*F*_(2, 66)_ = 37.74, *p* < 0.01, *η*^2^ = 0.533) (Fig. 3A) were revealed. Post-hoc analyses revealed significantly higher knee extension torque levels between the healthy control group and both primary and secondary ACL rupture groups (*p* < 0.01). Additional differences were found between patients with primary and secondary ruptures, with higher torque levels in the latter (*p* < 0.05).

#### Knee flexion torque

Regarding knee flexion torque in the injured leg, patients with primary ACL rupture demonstrated normalized torque levels of 1.01 ± 0.3 Nm/kg (76.7 ± 26.3 Nm) and patients with secondary ACL rupture torque levels of 1.14 ± 0.3 Nm/kg (85.7 ± 29.9 Nm). In the healthy control group, knee flexion torques of 1.59 ± 0.28 Nm/kg (125.0 ± 32.3 Nm) were observed. Calculations of one-way ANOVA revealed significant between-group differences (*F*_(2, 66)_ = 24.45, *p* < 0.01, *η*^2^ = 0.426), with significantly higher torque levels in the control group compared to both patients with a primary and secondary ACL rupture (*p* < 0.01) (Fig. 3B). No significant differences were observed between patients with primary and secondary ACL rupture (*p* = 0.14).

## Discussion

Primary and secondary ACL ruptures remain one of the predominate injuries in sports. The current study permits major insights into the force generating capacities of joint-stabilizing muscle groups prior to the arthroscopic ACL reconstruction. Significant group differences manifest a reduced maximal concentric torque of the knee extensors and flexors for patients with a primary and secondary ACL rupture in the injured and non-injured leg *before the* surgical reconstruction as compared to healthy matched controls. No significant differences were detected between patients with a primary as compared to patients with a secondary ACL rupture, except of knee extension on the injured leg.

### Pre-operative strength deficits in patients with primary and secondary ACL ruptures

Normalized isokinetic joint torque in the healthy population are 2.46 ± 0.44 Nm/kg in knee extension and 1.59 ± 0.28 Nm/kg in knee flexion which correspond to normative data which have been reported for non-athletic populations [39, 40]. It could have been expected that the values of the non-injured leg in patients with primary and secondary ACL rupture would correspond to the values of the healthy control group prior to surgery, but instead pronounced deficits have been found: strength deficits in the non-injured leg for knee extension were 21–22% and in knee flexion 22% for the primary and secondary ACL ruptures as compared to the healthy controls. Numerical diminutions in the leg with the torn ACL reached 36–44% for primary ACL ruptures and 28–31% for secondary ACL ruptures, respectively. This is a notable scientific finding as knee extensor and flexor torques serve as important shelters for articular knee structures protecting the ACL and compensating for the loss of articular stability or arthrokinematics dysfunction [41–43]. Knee extensors and flexors isometrically sustain forces up to 4500 N in dynamic movement [24] when the musculature is entirely innervated, whereas the ACL only sustains a force of approximately 400 N [23]. With a proportion of 1/10 of muscle force, the ACL is generally fable and its ligamentous integrity depends primarily on the stabilizing effects of the surrounding muscles [43]. The finding becomes particularly delicate in view of the healthy non-injured leg which shows likewise the strength deficits with equivalent magnitudes. As described by Wiggins et al. [10] secondary ACL ruptures are not limited to the operated limb. Recent evidence demonstrates, that rates of contralateral ACL injury exceed the rates of ipsilateral graft injury after ACLR, regardless of the graft side, age or activity level [10, 44]. It is supposed that increased incidences of contralateral injuries may be due to the persistence of the same risk factors that predispose patients to the initial injury which include significant strength deficits [30, 45]. Preoperative strength was measured at an average of 18 days after injury. It cannot be excluded that the patient still had pain or fear before the measurement which probably influenced the level of the measured torque values on both the injured and the non-injured leg. Nevertheless, the same prerequisites exist for both intervention groups, which allows its comparability.

### Differences between primary and secondary ACL ruptures

Scientific evidence exists which demonstrates that a multitude of biomechanical and muscular factors are predispositions for ACL injuries which underpins the clinical relevance of our data [46, 47]. Myer et al. [48] provide data on 205 young athletes and found an increased risk for ACL injury in athletes with an increased knee abduction moment, combined with reduced posterior chain strength. The combination of decreased relative hamstrings and high relative quadriceps strength is implicated as a potential mechanism for increased ACL injury [47–50].

Comparing patients before the primary to those before the secondary ACL revision, neither the knee extension nor the knee flexion revealed significant differences on the non-injured leg, but partially significant differences on the injured leg preferential the secondary revisions. Thus, although the strength of an ACL injured population is significantly lower than that of a healthy control group, deficits are independent of whether the patient has undergone a previous surgery or not. Our findings even favor patients with a history of ACL reconstruction in regard to the knee extensor strength which is elevated by 20% most probably due to consequent rehabilitation schemes. Previous studies on this issue are rare. The reason for this difference in strength between the two groups could be due to the patients’ greater awareness of pre- or rehabilitation and participation in exercise regimes to strengthen knee and provide balanced functional requisites prior to surgery [27]. This is in line with repeated measures studies analyzing the strength after ACL surgery at different time points. They demonstrate progressively increased torques from prior to surgery to the beginning of the rehabilitation up to one year [26, 51]. Riesterer et al. [26] found torque values for knee extension before surgery of approximately 1.3 Nm/kg pre-surgery and 1.6 Nm/kg 6-months post-surgery of the injured leg. It is noteworthy that exactly these values are in accordance to our data of the injured leg for primary, which correspond to pre-surgery values of patients suffering from a secondary ACL rupture and post-surgery values of patients after the primary ACL reconstruction, respectively. For the knee flexion the same scheme emerges with values of 1 Nm/kg pre-surgery and 1.1Nm/kg post-surgery corresponding to primary and secondary ruptures.

### Clinical implications

The present results are highly relevant and might help to support sports orthopaedical treatment and therapy in the prevention of ACL injuries.

First, systematically reduced strength in both primary and secondary ruptured individuals indicate that strength exercises and neuromuscular training interventions might be a relevant factor in reducing future risk of ACL injury. Although evidence from previous studies is conflicting regarding the direct effect muscle strength on injury prevention [52–54], lower quadriceps and hamstring strength may contribute to an increased risk of knee injury by facilitating instability in dynamic varus-valgus positions. In fact, timely identification of quadriceps weakness allows therapeutic interventions to specifically target neuromuscular competence and ensure optimization of postoperative outcomes [7]. The higher the strength before surgery, the higher it can be achieved after the surgery [26, 55]. However, it was beyond the scope of this article to investigate cause-effect relationships and remains to be shown in prospective studies whether the preoperative strengthening of thigh muscles or hip abductors will prevent secondary ACL ruptures or improve long-term outcomes.

Second and with regard to return to sports, injured/non-injured peak torque ratio (limb symmetry index (LSI)) is a widely used indicator in most return-to-sport algorithms [56, 57]. There is broad consensus that the side-to-side difference should not exceed ± 15% for healthy limbs, whereas differences beyond indicate a pathological state and insufficient prerequisites to return to sport [51]. However, in some sports there is asymmetry even in healthy athletes, which have indicated the necessity for individualized RTS criteria. Primary revisions in our study display a side-to-side deficit of 28% in knee extension and 19% in flexion, which is below the recommended values to participate in sports. In contrast, secondary revisions demonstrate lower deficits to the non-injured side with 13% in extension and 9% in flexion, which indicate a risk but not a pathological state [51]. Given that the present data implicate also pronounced decrements in muscular strength in the non-injured leg raises caution regarding the sole use of side-to-side symmetries when assessing the appropriate time point for RTS.

### Limitations

Besides matching all groups by age, sex and height/weight, both primary and secondary ACL patients received a standardized rehabilitation protocol (protocol published in [26]). However, physical activity outside the rehabilitation setting was not assessed in the current study. This might be considered when interpreting the present data. Although sex was included as a matching parameter, no in-depth analysis of gender-related differences was conducted in this study.

The patient group with primary ACL rupture was homogenized to the graft used for reconstruction (semitendinosus tendon). This population could not be matched to the secondary ACL group with respect to the graft as various graft sides were included in the first revision (Figs. 2 and 3), but only to age, sex and height/weight. However, since we focused on pre-operative values the graft choice of the primary ACL injury cannot have an influence in the primary ACL group. Moreover, for secondary ACL revisions the graft choice of the primary tendon was very individual, depending on concomitant injuries and patient history. Accordingly, the group of secondary ACL group is heterogeneous with regard to the primary tendon graft used in favor of homogeneity within their comorbidities. For this reason, only strength conditions before surgery—but not after surgery—were assessed in this study. After a secondary revision, very different outcomes with regard to strength conditions are to be expected due to the different tendon grafts and should be further investigated.

Finally, no conclusive statement can be drawn about cause-effect relationships of pre-operative muscular strength and injury risk since studies with longitudinal experimental designs are required. Nevertheless, the present data highlight the pronounced deficits in both knee extensor and flexor strength in primary and secondary ACL ruptured patients compared to healthy controls. Interestingly, these findings can also be observed on the non-injured site and are likely to predict functional outcomes following surgery as evidenced in previous studies [26].

## Conclusions

Patients—irrespective of primary or secondary ACL rupture—differ significantly in their strength prerequisites to their matched counterparts—on both the non-injured and the injured leg. However, there are hardly any differences between torques for patients with a secondary ACL as compared to a primary ACL rupture before their reconstruction surgery. Whether the observed decrements in muscular strength may contribute to an increased injury risk remains unclear and further prospective studies are warranted using predictive modelling approaches. Due to close associations between pre- and post-operative muscle strength evidenced in previous studies, the medical team should focus on a balanced strength and neuromuscular stabilization of the knee joint before and after a first revision of the ACL and also before a second revision.

## Data Availability

The datasets used and/or analysed during the current study are available from the corresponding author on reasonable request.
